# Real-world differences in denosumab persistence, reinitiation, and switching among cohorts of older adults in Canada and the United States

**DOI:** 10.1093/jbmrpl/ziaf061

**Published:** 2025-04-11

**Authors:** Kaleen N Hayes, Selvam R Sendhil, Sulbh Aggarwal, Andrew R Zullo, Sarah D Berry, Arman Oganisian, Michael Adegboye, Suzanne M Cadarette

**Affiliations:** Department of Health Services, Policy, and Practice, Center for Gerontology and Healthcare Research, Brown University School of Public Health, Providence, RI 02903, United States; Department of Epidemiology, Brown University School of Public Health, Providence, RI 02903, United States; Leslie Dan Faculty of Pharmacy, University of Toronto, Toronto, ON M5S 3M2, Canada; Department of Epidemiology, Brown University School of Public Health, Providence, RI 02903, United States; Leslie Dan Faculty of Pharmacy, University of Toronto, Toronto, ON M5S 3M2, Canada; ICES, Toronto, ON M4N 3M5, Canada; Department of Health Services, Policy, and Practice, Center for Gerontology and Healthcare Research, Brown University School of Public Health, Providence, RI 02903, United States; Department of Epidemiology, Brown University School of Public Health, Providence, RI 02903, United States; Hinda and Arthur Marcus Institute for Aging Research, Hebrew SeniorLife and Harvard Medical School, Boston, MA 02131, United States; Department of Medicine, Beth Israel Deaconess Medical Center, Boston, MA 02215, United States; Department of Biostatistics, Brown University School of Public Health, Providence, RI 02903, United States; Department of Health Services, Policy, and Practice, Center for Gerontology and Healthcare Research, Brown University School of Public Health, Providence, RI 02903, United States; Leslie Dan Faculty of Pharmacy, University of Toronto, Toronto, ON M5S 3M2, Canada; ICES, Toronto, ON M4N 3M5, Canada; Department of Public Health Sciences, Dalla Lana School of Public Health, University of Toronto, Toronto, ON M5T 3M7, Canada; Division of Pharmaceutical Outcomes and Policy, Eshelman School of Pharmacy, University of North Carolina, Chapel Hill, NC 27599, United States

**Keywords:** osteoporosis, denosumab, medication adherence, fractures, aged patients, bone density conservation agents, bisphosphonates, zoledronic acid

## Abstract

Denosumab is an injectable osteoporosis medication administered twice per year. Discontinuation of denosumab can result in rapid rebound fractures, but the evidence is limited on real-world persistence with denosumab. We conducted 2 parallel, population-based cohort studies leveraging (1) healthcare administrative data from Ontario, Canada (ON; 100% population) and (2) a 20% random sample of US Medicare beneficiaries (US). The first denosumab claim (US: 1/2010-12/2019; ON: 1/2012-12/2021) was identified using pharmacy claims (ON) and Medicare Parts D and B claims (US). Patients aged <66 yr, residing in long-term care (LTC), or with implausible data (eg, death before first claim) were excluded. We developed and applied an algorithm that used dosing and days between dispensations to clean denosumab claims. We assumed a days supply of 183 d for each dispensation and defined discontinuation as a 60-d gap in coverage. We estimated initial persistence, reinitiation, and switching to other osteoporosis medications using Kaplan–Meier estimators, censoring on death, disenrollment (US only), LTC admission, or study end (12/31/2022 [ON], 12/31/2020 [US]). We also estimated the monthly proportion of patients with an on-time denosumab dose to explore time trends. We identified 168 339 eligible individuals in ON (mean age = 78 yr; 90% female) and 97 595 in the US (mean age = 77 yr; 90% female). In ON, the median time to denosumab discontinuation was longer (median 2.3 yr [ON] vs 1.7 yr [US]; 3-yr persistence: 44% [ON] vs 31% [US]), and time to reinitiation was shorter (median = 0.5 yr [ON] vs 1.9 yr [US]). In both populations, around 10% switched to another osteoporosis medication. Women and those with prior oral bisphosphonate use had longer durations of denosumab treatment in ON but not in the US. The proportion persisting with on-time doses did not increase over time in the US or ON. Research to improve persistence with denosumab and optimize post-denosumab treatment is critical.

## Graphical Abstract

**Graphical Abstract:**
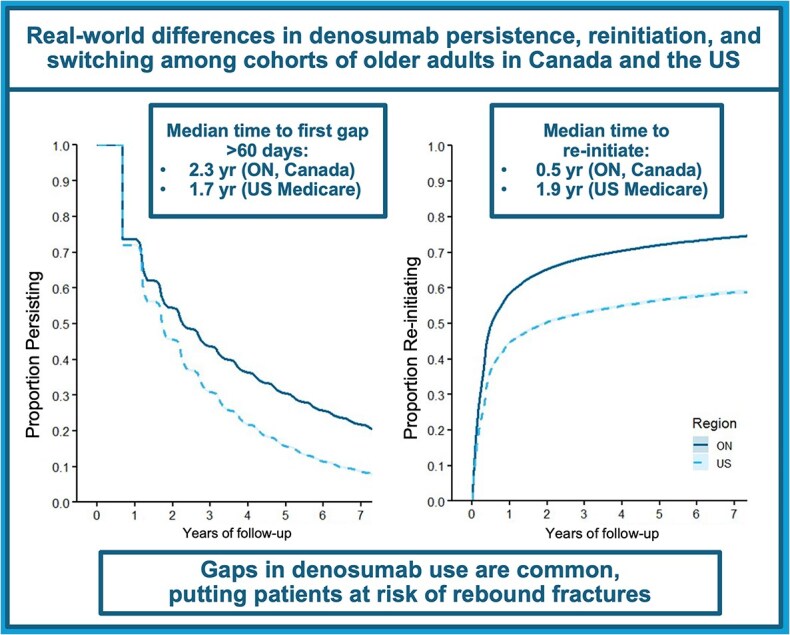

## Introduction

Denosumab is a monoclonal antibody administered every 6 mo for the treatment of osteoporosis. Denosumab prevents osteoclast maturation by blocking RANKL and reducing bone resorption and remodeling.^1^ Denosumab is effective at reducing the risk of vertebral and nonvertebral fractures.^2^ Uptake of denosumab has increased rapidly in both the US and Canada since it was first marketed in 2010,^3–5^ potentially due to its less frequent dosing compared to bisphosphonates, concerns about rare adverse events with bisphosphonates, and the longstanding question of the optimal length of treatment with bisphosphonates. However, recent post hoc analyses from clinical trials and real-world evidence have demonstrated that discontinuation of denosumab can result in rapid bone loss and rebound fractures.^6–8^ These fractures are likely the result of an increase in osteoclast maturation and activity immediately following the cessation of RANKL inhibition.^9^ Prior research suggests that rebound fractures can occur as early as 60 d after a missed dose.^10–12^ The degree of bone loss after denosumab discontinuation likely increases with longer therapy durations, and thus managing substantial bone loss after long-term treatment is a major challenge for clinicians.

Although reasons for missed doses and discontinuation have been documented (eg, side effects and delays from COVID-19 healthcare disruptions),^11^^,^^13^^,^^14^ persistence with denosumab, gap lengths among those who reinitiate denosumab, and switching from denosumab to other osteoporosis medications are unknown. The present study aimed to estimate and compare the length of initial persistence with denosumab treatment before a gap in therapy, the incidence and timing of reinitiation of denosumab, and incidence and timing of switching to other osteoporosis medications between older adults initiating denosumab in Canada and the US.

## Materials and methods

### Data sources

We completed 2 parallel, population-based cohort studies leveraging the following data sources: (1) healthcare administrative data for all residents of Ontario, Canada (ON data) and (2) US Medicare enrollment and claims data for a 20% random sample of beneficiaries (US data). In ON, all residents receive universal healthcare coverage for medically necessary services and procedures. Those aged 65 yr or older or on social assistance additionally receive coverage of drugs listed on the provincial formulary through the Ontario Drug Benefit (ODB). Other ON data sources included the Ontario Registered Persons Database for demographic and death data; the Canadian Institute for Health Information Discharge Abstract Database and National Ambulatory Care Reporting System for inpatient and emergency department visits, respectively; the Ontario Health Insurance Plan for outpatient physician services; and the Continuing Care Reporting System (CCRS) for long-term care (LTC) entry and services. These datasets were linked using unique encoded identifiers and analyzed at ICES.

US Medicare provides healthcare and prescription drug coverage for US residents aged 65 yr or older or with certain conditions. Medicare comprises Part D plans for prescription drugs and Fee-for-Service (Parts A and B, sometimes referred to as “traditional Medicare”) or Medicare Advantage (managed care) plans for medical services. We leveraged the Medicare Beneficiary Summary File for enrollment, demographic, and death data; Medicare Part D Event data for prescription drug dispensing claims; Medical Provider Analysis and Review files containing Medicare Part A data for hospitalizations and facilities use; Medicare Part B Carrier claims for outpatient services, including provider-administered medication claims; and the Residential History File,^15^ which leverages Medicare Part A facility and Part B claims to create a longitudinal record of patient residence in the community, post-acute care, LTC, and other settings. This study was approved by the Brown University Institutional Review Board, and the requirement for informed consent was waived (protocol #2022003400).

### Population and exposures

We identified all persons at their first claim for denosumab (60 mg) between January 1, 2012 and December 31, 2022 for ON and between January 1, 2010 and December 31, 2020 for the US. We identified denosumab use using pharmacy claims (ON) and Medicare Parts D and B claims (US). We then restricted the US data to those with ≥12 mo of Medicare Parts A, B, and D enrollment prior to the first denosumab claim (index date). For both cohorts, we then excluded patients with implausible data (eg, death date before index date), aged <66 yr on the index date, who used certain bone-related drugs in the 365 d prior to index or resided in LTC on the index date. Long-term care residence was identified using a flag in ODB claims and CCRS data for ON and the RHF for US data (only long-stay nursing home use [vs post-acute care] was considered an exclusion criterion). In ON, we additionally excluded those in hospital at the time of first denosumab use since claims arise only from community pharmacy dispensations (vs physician visit claims), and patients in ON subsequently take their denosumab with them to an outpatient visit.

Next, after empiric examination of denosumab claims, we developed and applied a denosumab claims data cleaning algorithm that used a combination of dosing, days between claims, and other administrative data. A detailed data cleaning algorithm and example SAS code are provided in the Supplementary Material (Table S6 and Figures S4-S7). In brief, using this algorithm, we flagged and excluded denosumab initiators with 3 or more claims sequentially occurring within 120 d because these patients were likely receiving denosumab 60 mg off-label for a cancer-related indication. Finally, we restricted inclusion to patients with an index date after December 31, 2021 (ON) or December 31, 2019 (US) to theoretically permit at least 12 mo of follow-up in each data source given our interest in describing persistence patterns. We measured patient characteristics using enrollment and claims data in a 12-mo lookback window (days 0-365 before the index date). Definitions used to define fractures in the lookback window are provided in Table S1.

### Outcomes

The primary outcome was length of persistence to denosumab, defining discontinuation of therapy as a 60-d gap in coverage. We imputed a value of 183 d covered per denosumab claim, which is the indicated dosing schedule for osteoporosis treatment.^16^ We also measured time to switch to another osteoporosis medication, defined as the first claim for an oral bisphosphonate (alendronate, risedronate, etidronate [ON only], or ibandronate [US only]), zoledronic acid, or anabolic treatment (abaloparatide [US only], teriparatide) any time after denosumab initiation. Romosozumab use was limited during the study period and thus not included. Raloxifene and calcitonin were excluded as they are sometimes used as add-on treatments to denosumab rather than as replacement therapy. Among those discontinuing, we measured time to reinitiate denosumab therapy (ie first denosumab claim after a discontinuation event).

### Follow-up and statistical analysis

Frequencies and proportions were reported to describe discrete patient characteristics, while means and SD were reported for continuous characteristics. Using Kaplan–Meier estimators, we quantified the median time to discontinuation of denosumab and the proportion persisting at prespecified time points (eg, 1-yr persistence); the proportion who reinitiated therapy and the median time to reinitiation, and the proportion starting another osteoporosis medication after denosumab initiation and the median time to switching. We followed patients forward until each respective event or censoring (death, LTC admission, disenrollment from Medicare, or study end [December 31, 2022 in ON, December 31, 2020 in US]). We also described the number of denosumab claims before discontinuation among those stopping therapy. In secondary analyses, we stratified discontinuation results by sex and history of oral bisphosphonate use in the year prior to denosumab initiation. Finally, to explore whether persistence improved in later years of the study as more evidence on rebound fractures became available, we examined trends in on-time dosing among patients persisting with denosumab treatment using methods similar to a prior analysis by Rzepka et al.^14^ We calculated and plotted the monthly proportion of patients with an on-time denosumab dose in both the US and ON data among the denosumab initiator cohorts. For both the US and ON cohorts, we leveraged data from January 1, 2015 to December 31, 2022 to capture the most recent trends in use. The number of patients due for a denosumab dose on each day of follow-up was based on the number of patients who received a denosumab dose 183 d earlier and who survived and remained uncensored and community-dwelling for 183 + 60 d after their prior denosumab dose. We then calculated the percentage of patients who received their denosumab dose “on time” (within ±60 d of the due date) for each calendar month. Because the study by Rzepka et al. demonstrated differences in the proportion with an on-time dose by duration of prior denosumab use, we calculated results stratified by whether the dose was due for a Novice User (due for second dose), Intermediate User (due for third or fourth dose), or Established User (due for ≥fifth dose) as of that month. We chose to describe trends qualitatively vs conducting a time-series analysis, as the purpose was to compare general trends in on-time dosing over time within and between ON and the US, rather than after a specific time point (eg, onset of the COVID-19 pandemic). Analyses were conducted in SAS version 9.4, SAS Enterprise Guide version 8.3, and R version 4.3.1.

## Results

### Study population

We initially identified 254 295 denosumab initiators in ON and 274 179 in the US. After applying exclusions and data cleaning restrictions (study exclusion diagrams in Figures S1 and S2), the final study sample comprised 168 339 eligible adults in ON (mean age = 78 [SD = 7.6] yr; 90% female) and 97 595 in the US (mean age = 77 [SD = 7.2] yr; 90% female). Table 1 presents baseline patient characteristics. The proportion of patients with a history of fracture in the year prior to initiating denosumab was similar between ON and the US, with 13.4% patients in ON vs 14.9% in the US having a recorded fracture of the vertebrae, radius/ulna, hip, pelvis, or humerus in the year prior to starting denosumab.

**Table 1:** Demographic and clinical characteristics of older adults initiating denosumab in Ontario, Canada (ON) and a sample of US Medicare Beneficiaries (US) (total *N* = 265 934).

	**ON (*N* = 168 339)**	**US (*N* = 97 595)**
**Age (yr), mean (SD)**	78.0 (7.5)	77.5 (7.2)
**Female, *N* (%)**	151 902 (90.2)	88 066 (90.2)
**Fractures in the year prior to denosumab initiation, *N* (%)**		
** Hip**	6446 (3.8)	2185 (2.2)
** Radius/ulna**	6423 (3.8)	2447 (2.5)
** Vertebrae**	6206 (3.7)	8490 (8.7)
** Pelvis**	5507 (3.3)	2022 (2.1)
** Humerus**	3476 (2.1)	1769 (1.8)
** Any of above**	22 559 (13.4)	14 543 (14.9)
**Osteoporosis medication use in the year prior to denosumab initiation (any), *N* (%)**		
** Any**	87 941 (52.2)	28 191 (28.9)
** Oral BP, *N* (%)**	86 233 (51.2)	21 008 (21.5)
** Alendronate**	26 095 (15.5)	15 469 (15.9)
** Risedronate**	60 426 (35.9)	2848 (2.9)
** Etidronate**	1621 (1.0)	N/A
** Ibandronate**	N/A	3435 (3.5)
** Other therapy, *N* (%)**	2614 (1.6)	8092 (8.3)
** Calcitonin**	25 (0.0)	1712 (1.8)
** Raloxifene**	2220 (1.3)	3065 (3.1)
** Teriparatide**	15 (0.0)	2401 (2.5)
** Zoledronic acid**	361 (0.2)	1106 (1.1)

Abbreviations: BP, bisphosphonate; ON, Ontario; SD, standard deviation; US, United States.

Fractures and prior therapies were measured in days −1 to −365 prior to index date.

N/A—ibandronate has not been marketed for osteoporosis in Ontario, and etidronate has not been marketed for osteoporosis in the US.

The ON cohort had nearly twice the proportion of patients with a history of osteoporosis medication use in the year prior to denosumab initiation compared to the US cohort (52.2% in ON vs 28.9% in the US). In ON, risedronate was the most common therapy used before denosumab (68.7% of those with prior treatment). Alendronate was most common in the US (54.9% of those with prior treatment). Prior use of other osteoporosis therapies (ie, raloxifene, teriparatide, calcitonin, and zoledronic acid) was less common in ON (1.6% of patients in ON vs 8.3% in the US).

### Length of initial persistence

**Figure 1:**
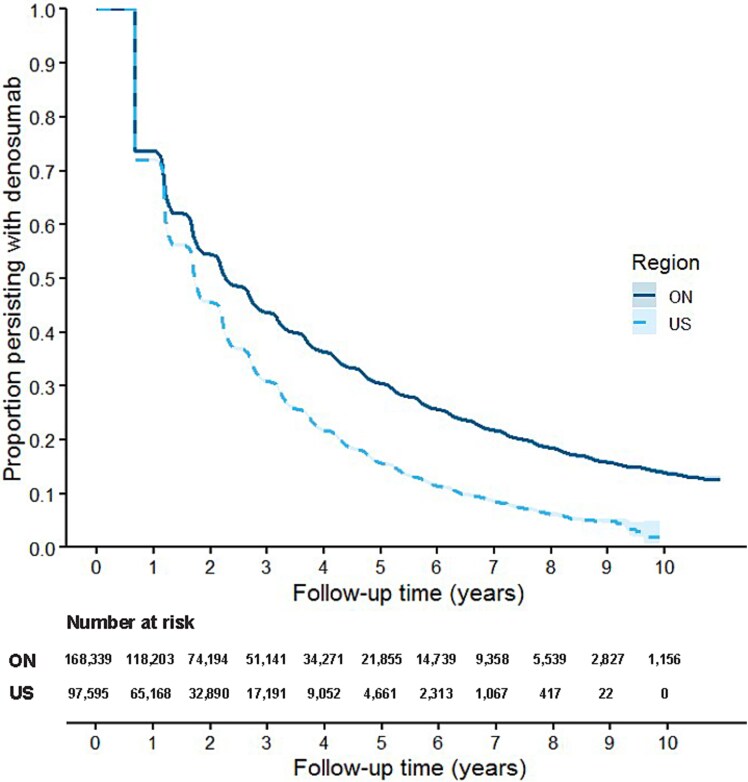
Initial persistence with denosumab among older adults in Ontario, Canada (ON) and a sample of Medicare beneficiaries (US), with 95% CIs. Censored on death, entry to LTC, disenrollment from Medicare (US only) or study end.

Kaplan–Meier curves presenting time to denosumab discontinuation in ON and the US are presented in Figure 1, and detailed estimates are provided in Table 2. At 1, 3, and 5 yr, the proportion of patients persisting with denosumab therapy in ON were 73.7%, 43.7%, and 30.5%, respectively. Likewise, the 1-, 3-, and 5-yr proportions persisting with denosumab in the US were 72.0%, 30.9%, and 15.7%, respectively. The median length of therapy (ie, time to discontinuation) was longer in ON (2.3 yr) compared to the US (1.7 yr). In both populations, most had 1 denosumab dose before discontinuation (40.6% in ON and 39.5% in the US; Table S2), but many had 5 or more doses before discontinuation (24.5% in ON and 18.2% in the US). The distribution of reasons for censoring is provided in Table S3.

**Table 2:** Kaplan–Meier results for proportion persisting with denosumab, reinitiating denosumab therapy after discontinuation, and switching to other osteoporosis medications in Ontario, Canada (ON) and a sample of Medicare beneficiaries (US).

	**Initial persistence** ^a^		**Reinitiating after 60-d gap** ^b^		**Starting new osteoporosis therapy** ^c^	
	**ON**	**US**	**ON**	**US**	**ON**	**US**
	**(*N* = 168 339)**	**(*N* = 97 595)**	**(*N* = 105 200)**	**(*N* = 65 818)**	**(*N* = 168 339)**	**(*N* = 97 595)**
**Time to event (yr), median**	2.3	1.7	0.5	1.9	Not reached	Not reached
**Proportion with event (%)**						
** 6 mo^d^**	N/A	N/A	50.0	37.1	4.9	3.4
** 1 yr**	73.7	72.0	58.5	44.7	6.2	4.7
** 3 yr**	43.7	30.9	68.5	53.1	9.2	9.0
** 5 yr**	30.5	15.7	72.1	56.6	11.2	12.8
** 10 yr**	13.9	2.0	76.4	59.5	14.6	19.4
**Censored, *N* (%)**	63 139 (37.5)	31 777 (32.6)	36 963 (35.1)	34 747 (52.8)	150 892 (89.6)	87 719 (89.9)

Abbreviations: ON, Ontario; US, United States.

a Proportion with event represents the proportion of people who persisted with denosumab (no gap >60 d).

b Proportion with event represents the proportion of people who reinitiated denosumab after discontinuation.

c Proportion with event represents the proportion of people who initiated new osteoporosis therapy (excluding raloxifene and calcitonin) any time after denosumab initiation.

d By design, no patient will have discontinued treatment at 6 mo due to 183 d of denosumab coverage and 60-d grace period.

Kaplan–Meier results for time to discontinuation stratified by sex and prior oral bisphosphonate use are presented in Table S4. In ON, the median length of therapy was longer for women than men: 2.3 vs 1.7 yr, respectively. In the US, the median length of therapy was 1.7 yr for both women and men, with similar proportions of persistence over time (eg, 1-yr persistence: 72.1% of women vs 70.8% of men). In ON, patients treated with oral bisphosphonates in the year prior to denosumab initiation (51.2%) had longer lengths of therapy than those without (2.7 vs 2.1 yr). However, in the US, the length of therapy was similar between those with vs without prior oral bisphosphonate use (median lengths of therapy 1.8 yr vs 1.7 yr).

### Reinitiating denosumab

**Figure 2:**
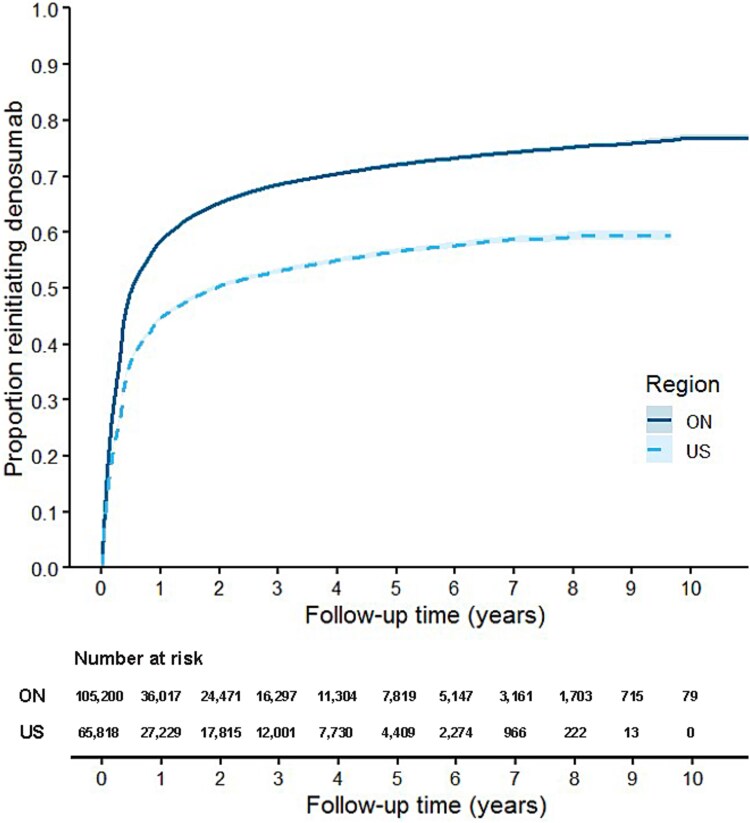
Time to reinitiate denosumab in older adults in Ontario, Canada (ON) and a sample of Medicare beneficiaries (US) who discontinued therapy, with 95% CIs. Censored on death, entry to LTC, disenrollment from Medicare (US only) or study end.

Among the 105 200 patients in ON who discontinued denosumab, 68 237 (64.9%) reinitiated therapy. In the US, 31 071 (47.2%) of the 65 818 patients who discontinued reinitiated denosumab. The median time to reinitiating denosumab was 0.5 yr in ON and 1.9 yr in the US (Figure 2). The proportion of patients reinitiating therapy at 1, 3, and 5 yr was higher in ON than in the US (58.5% vs 44.7%, 68.5% vs 53.1%, and 72.1% vs 56.6%, respectively; Table 2).

The proportions reinitiating denosumab stratified by sex and history of oral bisphosphonate use are presented in Table S5. Women had shorter times to reinitiate therapy than men (ie, median time to reinitiate denosumab: 0.5 yr for women and 0.9 yr for men in ON; 1.8 yr for women and 4.6 yr for men in the US). Those with prior treatment with oral bisphosphonates also had faster times to reinitiate therapy than those without (ie, median time to reinitiate: 0.4 yr for patients with bisphosphonate use vs 0.7 yr for others in ON; 1.3 yr for patients with bisphosphonate use vs 2.2 yr for others in the US).

### Treatment switching

There were 17 447 (10.4%) patients in ON and 9876 (10.1%) patients in the US who switched to another osteoporosis therapy after initiating denosumab, Table 2. Over a median follow-up time of 0.6 yr in ON, the 3 most common therapies among those who switched were risedronate (70.3%), alendronate (26.0%), and zoledronic acid (2.1%). In the US, over a median follow-up time of 1.2 yr, the 3 most common treatments among those who switched were alendronate (59.8%), zoledronic acid (12.8%), and ibandronate (12.3%).

### Monthly proportion with an on-time denosumab dose

**Figure 3:**
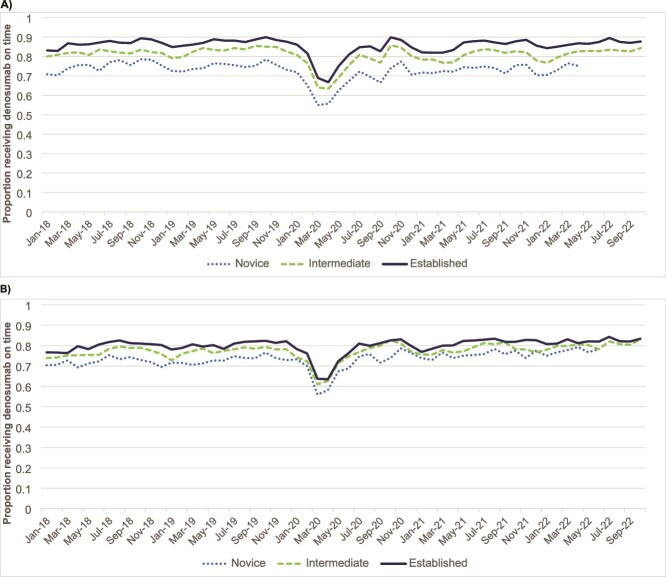
Proportion of older adults receiving denosumab therapy on-time in the community by month due, stratified by denosumab history in: (A) Ontario, Canada population; and (B) US Medicare sample (January 2018 to October 2022). Novice: due for second dose; intermediate: due for third or fourth dose; established: due for ≥ fifth dose. Figures displaying monthly data from January 2015 to Oct 2022 are available in the supplement.

Figure 3 presents the monthly proportions of patients due for a denosumab dose who received it on time (±60 d of the due date) in the ON and US cohorts. Because similar proportions with on-time dosing were observed in earlier years, these figures show results beginning in January 2018 (before the risk of rebound fractures after denosumab discontinuation became widely disseminated). Graphs with all data from 2015 to 2022 are available in Figure S3. Proportions for novice users in both cohorts are suppressed beyond April 2022 in ON and June 2022 in the US due to small cells.

In both cohorts, the proportions of patients persisting with therapy and with an on-time denosumab dose were similar in earlier vs later years. In both the ON and US cohorts, there was a reduction in on-time dosing in March and April 2020, coinciding with the onset of the COVID-19 pandemic. On-time dosing recovered in the US similar as in ON by June 2020. The overall average proportions with an on-time dose over the study period were higher in ON than the US (ON vs US: Novice Users 74% vs 72%; Intermediate Users 82% vs 76%; Established Users 86% vs 79%).

## Discussion

We conducted a multinational retrospective cohort study of denosumab persistence, reinitiation, and treatment switching among a total of 265 934 older adults initiating therapy in ON, Canada or enrolled in the US Medicare program. Denosumab was widely used in both ON and the US, and gaps in treatment were common. Specifically, by year 3 of treatment, less than half of patients in Ontario and less than a third in the US continued treatment. Around one-third of patients in both populations discontinued denosumab treatment after 4 or more doses (approximately 2 yr of therapy). We additionally conducted a serial cross-sectional analysis and observed no major increases in the proportion of patients persisting with denosumab and having on-time therapy in either ON or the US, even after the harms of gaps in denosumab use on rebound fractures were well-established.

We found that reinitiating denosumab therapy was relatively common, with around two-thirds in ON and half in the US reinitiating treatment, usually within 1 (ON) to 2 (US) yr after discontinuation. Only 13%-15% of patients in both populations in this study experienced a fracture in the year before denosumab initiation, and thus 10-yr fracture risk and bone mineral density may have served as the indication for starting treatment. Low initial persistence to medications used to treat an asymptomatic condition, such as osteoporosis without a fracture history, is common due to low perceived benefits of treatment.^17^ However, the short time to reinitiate suggests that the discontinuation of denosumab may occur due to unintentional gaps in treatment, rather than intentional stopping of therapy. Because rebound fracture risk can occur rapidly after a missed dose of denosumab, even short gaps in treatment may result in excess fracture risk. There is also limited evidence on the effects of reinitiating denosumab treatment on fracture risk; this clinical gap should be investigated to better understand the benefits of resuming denosumab therapy.

We identified notable differences in denosumab treatment patterns by country, sex, and history of other osteoporosis therapies. First, the median duration of denosumab therapy before discontinuation was longer in ON than in the US (2.3 vs 1.7 yr), and patients who discontinued therapy in ON also returned to therapy more quickly after a gap (average 0.5 vs 1.9 yr to re-start treatment among those who returned). Similarly, in the on-time dosing analysis, more patients in ON had an on-time dose than those in the US, and differences in persistence between the cohorts became larger with longer durations of denosumab therapy. The fragmentation of the US healthcare system, the varied coverage for denosumab provided by different Medicare Part D plans, and easier access to other treatment options like zoledronic acid or anabolics may have contributed to shorter lengths of therapy in the US. Professional bodies in Canada (eg, Osteoporosis Canada in October 2018)^18^ were also expeditious in releasing widespread statements about rebound fracture risk after denosumab discontinuation, which may have contributed to more vigilance among providers to prevent gaps in treatment. However, in neither country were major time trends identified in the proportion of patients with on-time denosumab dosing in later years of the study, despite far-reaching warnings about risks of abrupt discontinuation that can occur even after a short gap in denosumab use.

The length of denosumab therapy differed by sex and prior treatment with an oral bisphosphonate in ON but not in the US. Males had shorter lengths of denosumab therapy in ON compared to females. The reasons for the difference in persistence by sex in ON are unclear; however, although the overall proportion of males starting denosumab was similar in ON and US Medicare (around 10%), the use of denosumab in males has rapidly expanded in ON in recent years^5^ and is likely to surpass that in males in the US, which has remained relatively stagnant.^19^ Thus, these differences in persistence by sex between the countries may change. Second, those without bisphosphonate therapy prior to denosumab initiation discontinued denosumab more quickly than those with prior bisphosphonate use. Around half (52%) of those initiating denosumab treatment in ON had recent bisphosphonate use, compared to 29% in the US. For most patients, ON requires failure of or demonstrated intolerance to oral bisphosphonates prior to coverage of denosumab. Therefore, in ON, those who start denosumab de novo or after a long gap in bisphosphonate treatment likely have different characteristics and likelihood of persisting with treatment than those who transition directly from bisphosphonates. Conversely, in the US, denosumab may more commonly be implemented as an initial treatment. Patients starting denosumab vs oral bisphosphonates as initial therapy are therefore likely to be more similar and subsequently have comparable persistence with treatment. Importantly, given the long half-life of bisphosphonates, evidence on whether prior use of bisphosphonates can help protect against rebound fractures after denosumab discontinuation is critically needed.

Our study has notable strengths. We conducted a multinational examination of over 265 000 denosumab initiators, both male and female, to longitudinally examine patterns of use over time. Further, we implemented parallel methods, including a rigorous and transparent data cleaning algorithm for denosumab claims, that allowed for direct comparisons in denosumab use between US Medicare beneficiaries and older adults in Ontario, Canada. This algorithm improved upon traditional methods of using denosumab claims by correcting for errors in days supply values and excluding Medicare Part B claims for denosumab that occurred at intervals consistent with malignancy-based dosing, because no specific codes exist for denosumab use in malignancy vs osteoporosis presently. However, our study had potential limitations. We were limited to data from one Canadian province and that from US FFS Medicare beneficiaries. Denosumab discontinuation rates may differ in patients in other Canadian provinces or Medicare Advantage beneficiaries due to differences in insurance coverage or healthcare access for denosumab administration. Finally, though we implemented a rigorous data cleaning algorithm, we cannot say with certainty that we excluded all use for cancer, though any residual use in the cohort is likely rare.

In conclusion, among 168 339 older adults in Ontario and 97 595 Medicare beneficiaries in the US, we estimated the average treatment length of denosumab was around 2 yr, with longer average treatment in ON. Some patients reinitiated therapy, and a small number switched to other treatments, but many patients appeared to stop denosumab without any replacement osteoporosis therapy. Further research on the optimal strategies to prevent fractures after denosumab discontinuation, including transition to other osteoporosis therapies or pretreatment with bisphosphonates, is critically needed to guide clinical decision-making for osteoporosis.

## Supplementary Material

supplement_dmab_dc_jbmrplus_R1_250320sub_ziaf061

## Data Availability

Data from this study are used under strict terms of data use agreements from the Centers for Medicare and Medicaid Services and ICES. Sharing of data is strictly prohibited under these data use agreements. Software code related to denosumab data cleaning is provided in the supplemental materials, and additional software code may be provided upon reasonable request.
