# Supplementary material for: Real-world differences in denosumab persistence, reinitiation, and switching among cohorts of older adults in Canada and the United States
Source: JBMR Plus. 2025 Apr 11;9(6):ziaf061. doi: 10.1093/jbmrpl/ziaf061 (PMC12087952; doi:10.1093/jbmrpl/ziaf061)
Supplement: supplement_dmab_dc_jbmrplus_R1_250320sub_ziaf061 [file supplement_dmab_dc_jbmrplus_r1_250320sub_ziaf061.docx]

**Table S1.** Fracture definitions used in 12-month lookback from index date (first denosumab claim)

**Table S2.** Number of denosumab dispensations before discontinuation

**Table S3.** Reasons for censoring— initial denosumab persistence analysis

**Table S4.** Kaplan-Meier results for initial persistence stratified by (a) sex and (b) prior oral bisphosphonate use

**Table S5.** Kaplan-Meier results for time to reinitiate denosumab after discontinuation stratified by (a) sex and (b) prior oral bisphosphonate use

**Figure S1.** Exclusion flow diagram for Ontario, Canada population (ON)

**Figure S2.** Exclusion flow diagram for US Medicare population (from 20% random sample)

**Figure S3.** Proportion of patients receiving denosumab therapy on-time in the community by month due, stratified by denosumab history in a) Ontario, Canada population; and b) US Medicare sample, (Jan 2015 - Sept 2022).

**DENOSUMAB CLAIMS DATA CLEANING ALGORITHM**

**Table S6.** Definitions for study drug inclusion and exclusion

**Figure S4.** Exposure data cleaning steps

**Figure S5.** Summary of “<121 data cleaning rules” for illogical denosumab dispensations

**Figure S6.** Data cleaning algorithm example

**Figure S7.** Example SAS code for implementing data cleaning of denosumab claims in Medicare data.

**Table S1.** Fracture definitions used in 12-month lookback from index date (first denosumab claim)

| **Fracture type** | **Codes*** |
| --- | --- |
| Hip | **ICD-9:**  820 (Fracture of neck of femur)  **ICD-10:**  S72.0 (Fracture of head and neck of femur)  S72.1 (Pertrochanteric fracture)  S72.2 (Subtrochanteric fracture of femur) |
| Humerus | **ICD-9 and OHIP (ON only):**  812 (Fracture of humerus)  **ICD-10:**  S42.2 (Fracture of upper end of humerus)  S42.3 (Fracture of shaft of humerus)  S42.4 (Fracture of lower end of humerus) |
| Radius/ulna | **ICD-9 and OHIP (ON only):**  813 (Fracture of radius and ulna)  **ICD-10:**  S52.x (Fracture of forearm) |
| Pelvis | **ICD-9 and OHIP (ON only):**  808 (Fracture of pelvis)  **ICD-10:**  S32.1 (Fracture of sacrum)  S32.3 (Fracture of ilium)  S32.4 (Fracture of acetabulum)  S32.5 (Fracture of pubis)  S32.7 (Multiple fractures of lumbar spine and pelvis)  S32.8 (Fracture of other and unspecified parts of lumbar spine and pelvis) |
| Vertebrae | **ICD-9 and OHIP (ON only):**  805 (Fracture of spine and trunk)  **ICD-10**:  S22.0 (Fracture of thoracic vertebra)  S22.1 (Fracture of first thoracic vertebra)  S32.0 (Fracture of lumbar vertebra)  S32.7 (Multiple fractures of lumbar spine and pelvis)  S32.8 (Fracture of other and unspecified parts of lumbar spine and pelvis) |

*ICD: International Classification of Diseases; OHIP – Ontario Health Insurance Plan outpatient billing codes; ON – data from Ontario, Canada.*

**Inpatient (hospitalizations) and all diagnostic coding positions (e.g., primary, first secondary) were considered for all fractures except hip fractures. For hip fractures, only inpatient claims or emergency department claims were considered, but all diagnostic positions were evaluated for codes. All subcodes under the specified diagnostic parent code were included (e.g., “S72.2” encompasses all subcodes like S72.21, S72.22XA, etc.)*

**Table S2.** Number of denosumab dispensations before discontinuation

| **Number of dispensations before discontinuation, N (%)** | **ON**  (N=105,200) | **US**  (N=65,818) |
| --- | --- | --- |
| 1 | 42,737 (40.6%) | 26,007 (39.5%) |
| 2 | 18,028 (17.1%) | 13,733 (20.9%) |
| 3 | 10,832 (10.3%) | 8,187 (12.4%) |
| 4 | 7,799 (7.4%) | 5,938 (9.0%) |
| 5+ | 25,804 (24.5%) | 11,953 (18.2%) |
| *ON: Ontario; US: United States.*  *Discontinuation defined by minimum 60-day gap.* | | |

**Table S3.** Reasons for censoring— initial denosumab persistence analysis

| **Censoring reason** | **ON, N (%)**  (N=168,339)  *Median follow-up: 2.3 yr*  *Mean follow-up: 2.5 yr* | **US, N (%)**  (N=97,595)  *Median follow-up: 1.7 yr*  *Mean follow-up: 2.3 yr* |
| --- | --- | --- |
| Administrative  (i.e., data end date) | 44,498 (26.4%) | 18,413 (18.9%) |
| Death | 13,129 (7.8%) | 5,716 (5.9%) |
| Long-term care entry | 5,512 (3.3%) | 711 (0.7%) |
| Medicare disenrollment | -- | 6,937 (7.1%) |
| *ON: Ontario; US: United States.*  *Note: Data end date was 31-Dec-2022 in ON and 31-Dec-2020 in the US. Mean follow-up was approximated using Kaplan-Meier survival estimates as the weighted average:* $\sum\left( {time}_{years}*survival\_probability \right)/ \sum(survival\_probability)$*where time_years_ represents follow-up time, and survival_probability denotes the corresponding survival probabilities at those times. This approach weights each time point by the probability of survival, providing an approximation of the average follow-up duration.* | | |

**Table S4.** Kaplan-Meier results for initial persistence stratified by (a) sex and (b) prior oral bisphosphonate use

|  | **ON**  N=168,339 | | **US**  N=97,595 | |
| --- | --- | --- | --- | --- |
| 1. **Sex stratification** | **F** | **M** | **F** | **M** |
|  | n=151,902 | n=16,437 | n=88,066 | n=9,529 |
| **Time to event (years), median** | 2.3 | 1.7 | 1.7 | 1.7 |
| **% persisted** |  |  |  |  |
| 1 year | 74.2% | 69.2% | 72.1% | 70.8% |
| 3 years | 44.6% | 34.8% | 31.1% | 28.4% |
| 5 years | 31.2% | 23.4% | 15.9% | 13.8% |
| **Censored, N (%)** | 56,342 (37.1%) | 6,797 (41.4%) | 28,367 (32.2%) | 3,410 (35.8%) |
|  |  | |  | |
| 1. **Prior oral BP use** | **Yes** | **No** | **Yes** | **No** |
|  | n=86,233 | n=82,106 | n=21,008 | n=76,587 |
| **Time to event (years), median** | 2.7 | 2.1 | 1.8 | 1.7 |
| **% persisted** |  |  |  |  |
| 1 year | 76.8% | 70.5% | 75.0% | 71.2% |
| 3 years | 47.0% | 40.2% | 33.8% | 30.1% |
| 5 years | 33.1% | 27.8% | 17.4% | 15.3% |
| **Censored, N (%)** | 33,364 (38.7%) | 29,775 (36.3%) | 7,245 (34.5%) | 24,532 (32.0%) |
|  | | | | |
| *BP: bisphosphonate; ON: Ontario; prop: proportion; US: United States.* | | | | |
| *Note: Prior oral BP use was measured within 365 days before denosumab initiation.* | | | | |

**Table S5.** Kaplan-Meier results for time to reinitiate denosumab after discontinuation stratified by (a) sex and (b) prior oral bisphosphonate use

|  | **ON**  N=105,200 | | **US**  N=65,818 | |
| --- | --- | --- | --- | --- |
| 1. **Sex stratification** | **F** | **M** | **F** | **M** |
|  | n=95,560 | n=9,640 | n=59,699 | n=6,119 |
| **Time to event (years), median** | 0.5 | 0.9 | 1.8 | 4.6 |
| **Proportion reinitiated** |  |  |  |  |
| 1 year | 59.2% | 51.5% | 45.1% | 40.5% |
| 3 years | 69.1% | 63.1% | 53.6% | 47.6% |
| 5 years | 72.4% | 69.5% | 57.1% | 50.6% |
| **Censored, N (%)** | 32,793 (34.3%) | 4,170 (43.3%) | 30,684 (51.4%) | 3,564 (58.2%) |
|  |  | |  | |
| 1. **Prior oral BP use** | **Yes** | **No** | **Yes** | **No** |
|  | n=52,869 | n= 52,331 | n=13,763 | n=52,055 |
| **Time to event (years), median** | 0.4 | 0.7 | 1.3 | 2.2 |
| **Proportion reinitiated** |  |  |  |  |
| 1 year | 61.8% | 55.1% | 47.9% | 43.8% |
| 3 years | 71.3% | 65.7% | 56.5% | 52.1% |
| 5 years | 74.6% | 69.6% | 60.1% | 55.6% |
| **Censored, N (%)** | 17,019 (32.2%) | 19,944 (38.1%) | 6,686 (48.6%) | 27,562 (53.0%) |
|  | | | | |
| *BP: bisphosphonate; ON: Ontario; prop: proportion; US: United States.* | | | | |
| *Note: Prior oral BP use was measured within 1 year before denosumab initiation.* | | | | |

**Figure S1.** Exclusion flow diagram for Ontario, Canada population (ON)

**
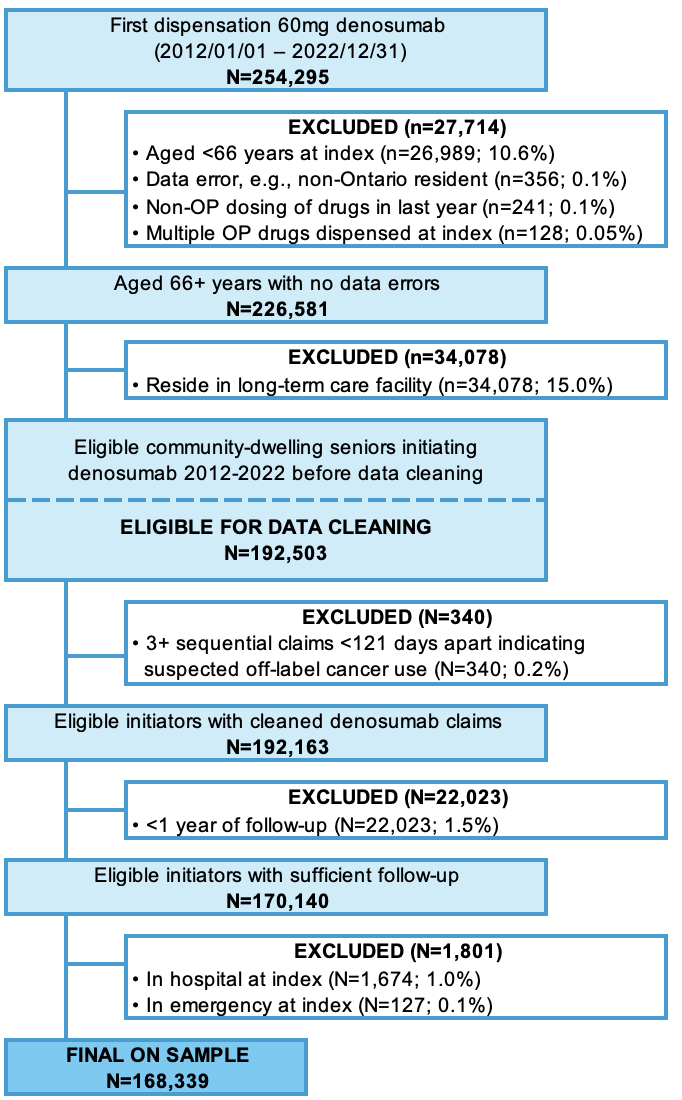
**

*Emergency: Emergency Department; ON: Ontario; OP: osteoporosis.*

**Figure S2.** Exclusion flow diagram for US Medicare population (from 20% random sample)

**
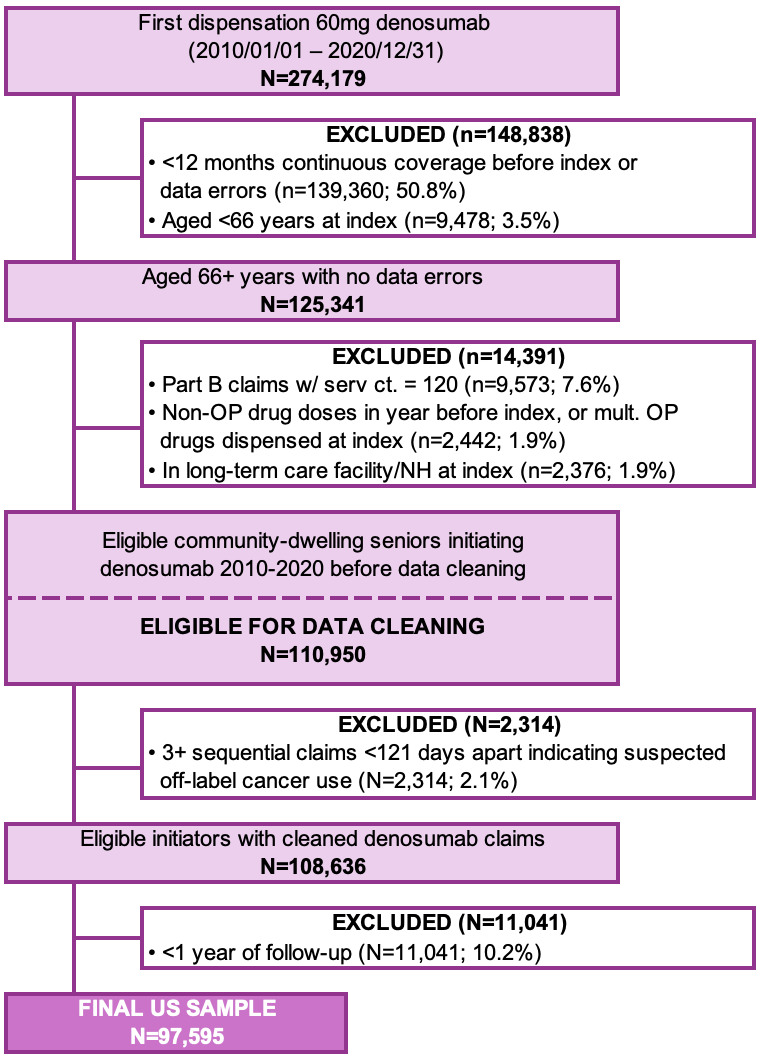
**

*OP: osteoporosis; US: United States.*

**Figure S3.** Proportion of older adults receiving denosumab therapy on-time in the community by month due, stratified by denosumab history in a) Ontario, Canada population; and b) US Medicare sample, (Jan 2015 – Oct 2022).

*Novice: due for 2^nd^ dose; intermediate: due for 3^rd^ or 4^th^ dose; Established: due for ≥5^th^ dose.*

1. ON data
2. **US data**

**DENOSUMAB CLAIMS DATA CLEANING ALGORITHM**

**OVERVIEW**

This document supplements information provided in the main manuscript by detailing: 1) study flow for inclusion, and 2) exposure data cleaning methods.

**Study Flow (Inclusion / Exclusion)**

We previously identified first dispensation date of any osteoporosis drug, as well as separate initiation cohorts for each osteoporosis medication (alendronate, etidronate, ibandronate, risedronate, nasal calcitonin, denosumab, raloxifene, abaloparatide, teriparatide, zoledronic acid and romozosumab) in Ontario (ON) using the ON Drug Benefit (ODB) data from 1996 to 2022, and data from a 20% random sample of Medicare beneficiaries from 2008 to 2020 (United States, US).^1^ **Table S6** provides definitions for eligible and ineligible formulations and doses of osteoporosis medications. Persons dispensed more than a single type of osteoporosis medication at index were excluded since practice guidelines encourage starting with monotherapy, and thus receiving multiple different osteoporosis medication at index is likely a marker of incomplete drug history.

In the current study, we focus on persons initiating denosumab (60 mg). We clarify here specific approaches used to identify eligible denosumab dispensation in ON vs US. All drugs in Canada receive a unique Drug Identification Number (DIN) upon Notice of Compliance to market the drug, and each DIN is specific to strength.^2^ At the time of analysis, only two DINs were available for denosumab: 02343541 for 60 mg (included), and 02368153 for 120 mg (excluded). In the US Medicare data, UpToDate® Lexidrug™ (formerly Lexicomp®) was used to identify denosumab claims in the Part D Event file using generic name variables generated from National Drug Codes. Generated dose variables were then used to include denosumab 60 mg and exclude denosumab 120 mg. The Medicare Part B line file that provides additional for corresponding Part B claims was used to identify claims for denosumab administration. Line items for Healthcare Common Procedure Coding System code J0897 were identified. Because J0897 captures both 60 mg and 120 mg claims, we explored the Part B line file’s “service count” variable, which took the values of 1, 60, or 120, or other (<0.3% of all Part B claims). The service count variable is specific to Part Medicare B claims and is defined by the Centers for Medicare and Medicaid Services Research Data Assistance Center as *“The count of the total number of services processed for the line item on the non-institutional claim.”* ^3^ In preliminary analysis, we noted that the service count variable often had values corresponding to denosumab doses (60 or 120). Further, we found that patients with a service count equal to 120 at index had shorter refill lengths (median time to next claim [Q1, Q3] = 28 [28, 35]). We interpreted service count=1 as 1 unit rather than the mg of the medication being dispensed, as the median time to next claim was 183 days for claims with this service count value. We used this value to inform exclusion criteria, below.

Denosumab was first available through ODB program in February 2012, and through Medicare in January 2010. We included all 60 mg denosumab dispensation claims (DIN=02343541) submitted to ODB through to December 31, 2022, and considered Medicare Part D and B claims through to December 31, 2020. Here we identified 254,295 eligible persons in ON, and 274,179 in the US. **Figures S1 and S2** present the study flow of eligible patients, resulting in 168,339 eligible adults in ON (**Figure S1**) and 97,595 in US (**Figure S2**). Patients were first excluded due to data errors (e.g., death date prior to index, missing age) or incomplete coverage (ON: excluded non-residents, US: restricted to those with ≥12 months of Medicare Parts A, B, and D enrollment prior to index date). We next excluded people with use of non-osteoporosis dosing of osteoporosis drugs as well as the bisphosphonates clodronate and pamidronate in the 0 to 365 days prior to index, multiple osteoporosis drugs at index as an indicator of incomplete drug history, and patients aged <66 years at index. In the US, patients with Part B claims associated with a service count of 120 were removed. Of remaining, 34,078 (13.4%) in ON and 2,376 (1.9%) in the US residing in long-term care institutions were excluded. The remaining 192,503 in ON and 110,950 in USA were eligible to undergo exposure (denosumab) data cleaning. Finally, we restricted inclusion to patients initiating denosumab (first claim) until December 31, 2021 (ON) or December 31, 2019 (US) to theoretically permit at least 1 year of follow-up given our interest in describing adherence patterns.

A post hoc decision was made in ON to exclude patients in hospital or emergency and not discharged to community at index (<0.1%); given that only outpatient pharmacy claims are available and denosumab claims through ODB are triggered when a pharmacist fills the medication (dispensing data), and not when a patient picks up their prescription. Thus, patients with dispensation record while in-hospital and not discharged to community would not be community-dwelling and thus not eligible for inclusion.

**Denosumab Exposure Data Cleaning**

This section describes the steps and rationale for initial data analysis and exposure data cleaning for denosumab. Denosumab is a subcutaneous injection dosed semi-annually (every 6 months) for the treatment of osteoporosis. In Ontario, patients historically submitted their prescription to a community pharmacy, the pharmacy dispenses the medication, and the patient subsequently brings the medication to a clinic appointment to be administered by a physician or nurse.^4^ However, following the COVID-19 pandemic, patients were given the option to self-administer denosumab following education and training. Still, most patients receive denosumab during a clinical appointment and if a patient changes their clinical appointment, they will logically wait to pick-up denosumab until closer to their clinical appointment. Similarly, if a pharmacist submits a claim with an error, they may retract the claim and resubmit corrected on the same date, or within the next subsequent days. While claim retractions due to reshelving a medication or resubmitting due an error should be cleaned, i.e., the original claim retracted should not appear in the ODB database, exceptions occur.

In addition, while a 6-month interval is recommended, it is possible that a patient will receive an early dose. Upon consultation with clinical experts, we reached consensus that a patient should not receive two doses of denosumab within 120 days (4 months) for osteoporosis. Any earlier timing could be indicative of off-label use for cancer, or errors due to claim retractions that appear as valid dispensations. The initial steps before applying our data cleaning rules were to 1) delete ineligible claims (duplicate claims that occur on the same day, claims that occur after death, claims occurring in long-term care), 2) delete ineligible people (≥3 denosumab claims in a row <121 days apart), and then identify and focus on cleaning claims for all remaining people with at least two denosumab claims < 121 days apart, the maximum number among remaining eligible people.

We thus developed “<121 days data cleaning rules,” to clean denosumab claims for these patients, illustrated in **Figure S5**. Henceforth, we refer to any claim occurring <121 days after the prior claim as an “illogical” claim and claims occurring ≥121 days after the prior as “logical.” We applied different strategies depending on whether there were one versus two illogical claims in a row. When one claim was illogical, we deleted the prior claim and used the previously illogical claim date as the true date (**Figure S5a**). For example, if a claim occurred 7 days after the index claim, the original index claim was deleted, and the new index date was adjusted by 7 days. When two claims were illogical, data cleaning steps depended on the sum of the number of days between the claims. If the sum of the number of days between the illogical claims was less than 121 (e.g., the first illogical claim occurred 35 days after the prior claim, then the second illogical claim occurred 37 days thereafter), we retained only the second “illogical” claim and deleted the prior two claims (**Figure S5b**). Finally, if the sum of the number of days between the illogical claims was greater than 121, we deleted the first illogical claim (**Figure S5c**). Another example application of the data cleaning algorithm is provided in **Figure S6**.

**REFERENCES**

1. Hayes, KN, Aggarwal, S, Cadarette, SM. Trends in osteoporosis drug initiation and use among community dwelling older adults. *Pharmacoepidemiol Drug Saf*. 2023;32(S1):363. doi:10.1002/pds.5686

2. Canada H. Notice of Compliance (NOC) Database. May 12, 2005. Accessed December 4, 2024. https://www.canada.ca/en/health-canada/services/drugs-health-products/drug-products/notice-compliance/database.html

3. ResDAC. Line Service Count (FFS). https://resdac.org/cms-data/variables/line-service-count-ffs

4. Rzepka AM, Cheung AM, Kim S, Gomes T, Cadarette SM. On-time denosumab dosing recovered rapidly during the COVID-19 pandemic, yet remains suboptimal. *JBMR Plus*. 2024;8(5):ziae027. doi:10.1093/jbmrpl/ziae027

**Table S6.** Definitions for study drug inclusion and exclusion

| **Medication** | **Eligible** | **Ineligible**  **(excluded based on 1-year lookback from index)** |
| --- | --- | --- |
| **Exposure of interest** |  |  |
| Denosumab | ON:*  60 mg  US:  Part D:** 60 mg  Part B: J0897, service count ≠ 120 | ON:  120 mg  US:  Part D:** 120 mg  Part B: J0897, service count = 120 |
| **Osteoporosis medication history***** |  |  |
| Alendronate | 5 mg, 10 mg, 35 mg, 70 mg | 40 mg |
| Etidronate (cyclical with calcium – ON only) | 400 mg with 500 mg or 1250 mg calcium carbonate | 50 mg, 200 mg, 400 mg |
| Ibandronate (US only) | 2.5 mg, 150 mg | 3 mg/3 mL |
| Risedronate | 5 mg, 35 mg, 75 mg, 150 mg | 30 mg |
| Raloxifene | 60 mg | n/a |
| Nasal calcitonin | 200 IU / spray | all other formulations |
| Abaloparatide (US only) | All formulations | N/A |
| Romosozumab (US only) | All formulations | N/A |
| Teriparatide | 250 mcg / ml | N/A |
| Zoledronic acid | 5 mg / 100 ml | 4 mg / 5 ml |
| Other bisphosphonates |  |  |
| Clodronate | n/a | all formulations |
| Pamidronate | n/a | all formulations |

**ON: DIN= 02343541 (60 mg), 02368153 (120 mg)*

**US: 60 mg NDC= 55513-0710 (all packages), 120 mg NDC= 55513-0730-01*

****eligible doses also considered as part of switch during analyses that looked forward from index*

**Figure S4.** Exposure data cleaning steps

**
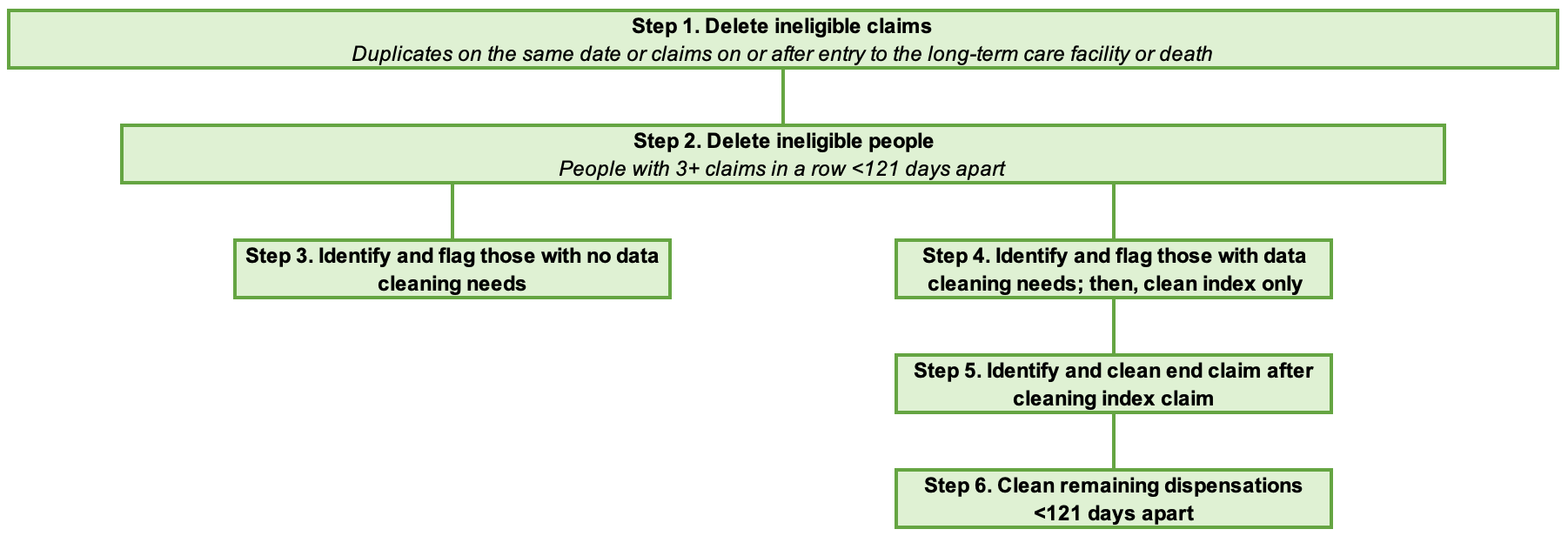
**

**Figure S5.** Summary of “<121 data cleaning rules” for illogical denosumab dispensations

7 days apart, respectively. Applying the 121 day data cleaning rule,

Consensus identified any two denosumab claims within 120 days as “illogical” and subject to the following data cleaning rules. We developed different strategy when there was one illogical claim vs. two illogical claims in a row. Simple examples are provided below.

1. **When one illogical (<121 days after prior claim) claim identified:**

delete first claim and move to next (“added to prior”)

- - delete the first claim since next claim < 121 days later, and use the next claim date as true date
  - In this example, the new index date is seven days after the original index claim date (cleaned index date = claim 1 date)


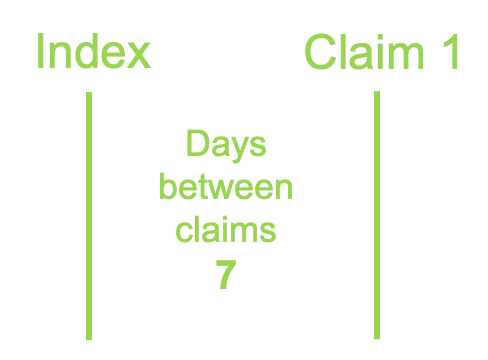


1. **When two illogical claims in a row, and adding the days between the three claims is:**
2. **< 121:** days between first and third claims add to less than 121
   - delete first two claims and keep the third date as true date
   - In this example, the new index date is 72 (35+37) days after the original index claim date (cleaned index date = claim 2 date)

**
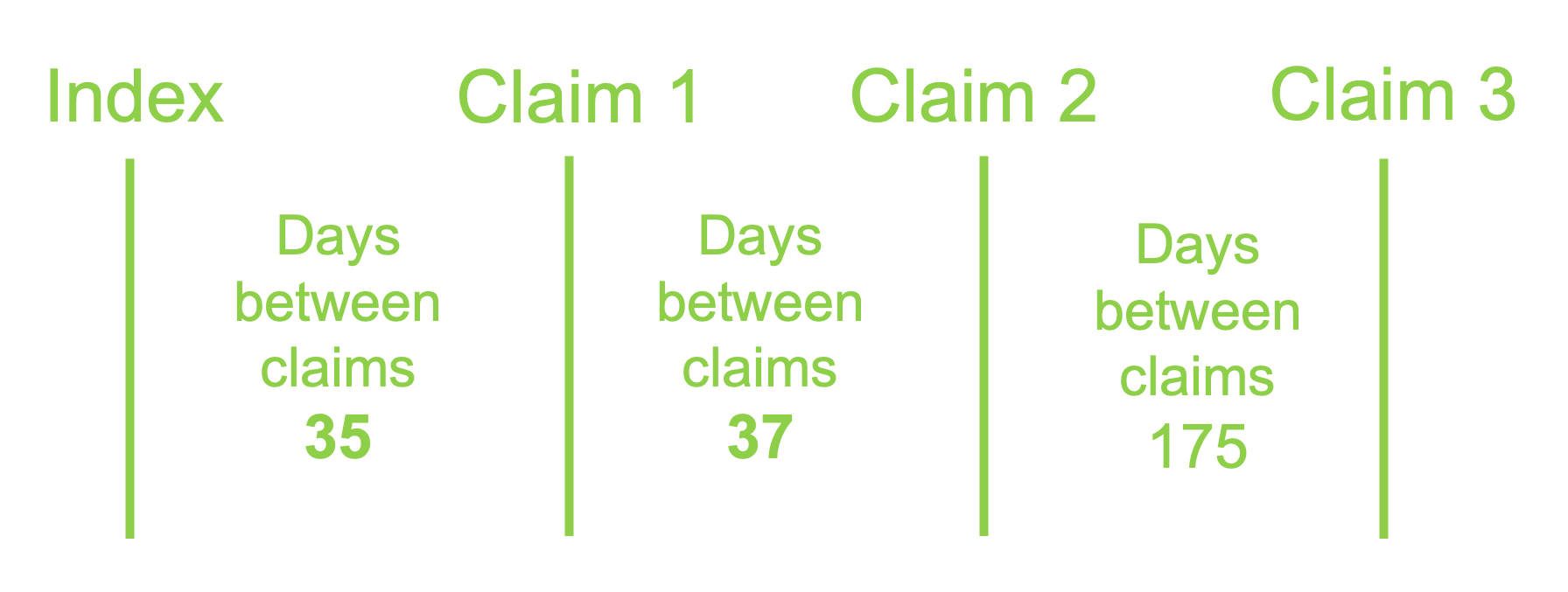
**

1. **≥ 121**: days between first and third claims add to ≥121
   - keep first claim date, then add subsequent two claims together as next true date
   - In this example, there is no change to the index date, Claim 1 is deleted, and Claim 2 is retained


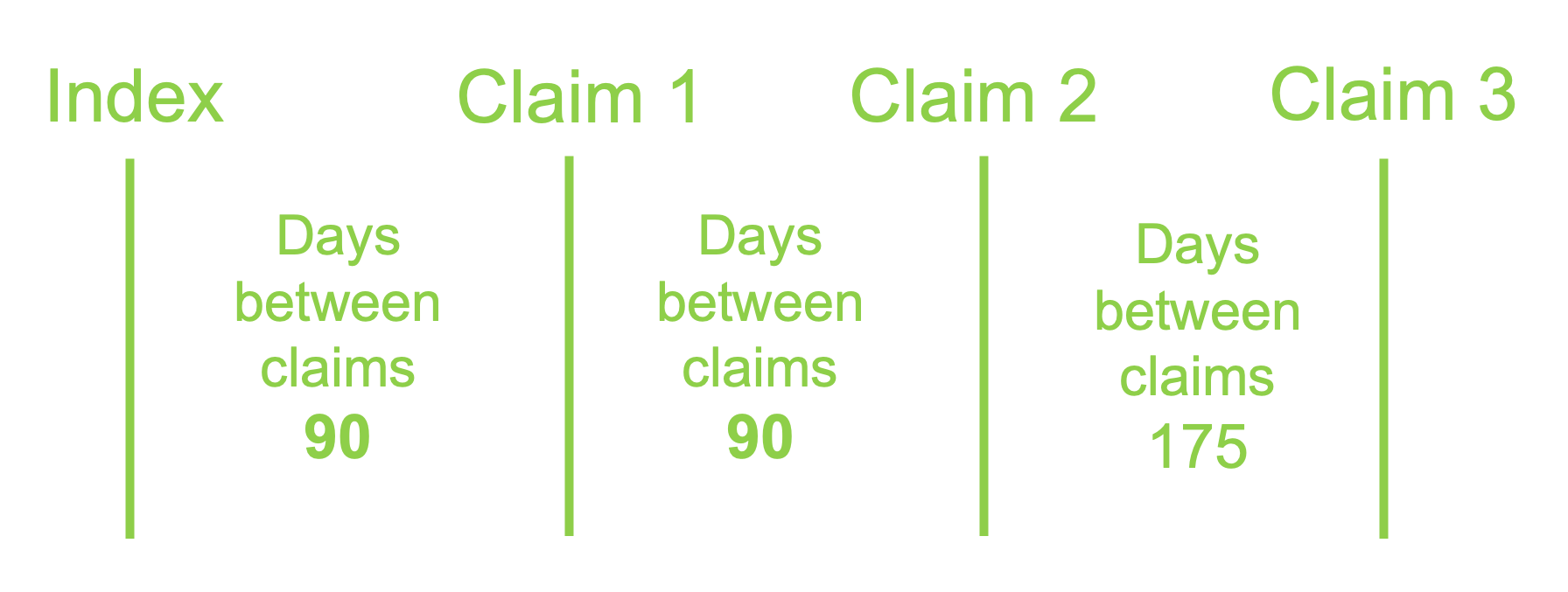


**Figure S6.** Data cleaning algorithm example


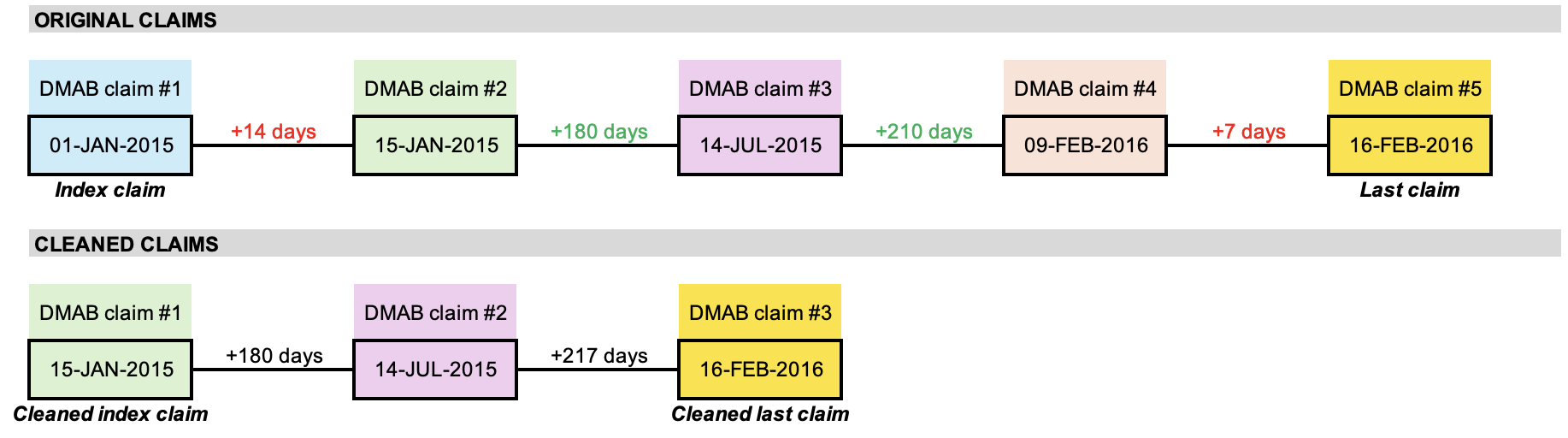


*DMAB: denosumab*

**Figure S7.** Example SAS code for implementing data cleaning of denosumab claims in Medicare data.

/************************************************************************

*PROGRAM NAME: Denosumab Claims Cleaning Algorithm;

*CREATED BY: Selvam Sendhil, Michael A. Adegboye, Kaley (Kaleen) Hayes;

*ASSOCIATED MANUSCRIPT: Real-world differences in denosumab persistence, reinitiation and switching

among cohorts of older adults in Canada and the United States (Hayes et al.)

*CREATION DATE: 16-DEC-2024;

*LAST UPDATED: 12-MAR-2025;

*GENERAL PURPOSE: Create a cleaned dataset of denosumab claims for

osteoporosis from Medicare Parts B and D;

*INPUT DATASET:

1. DMAB_CLAIMS: *Dataset containing all denosumab claims from Medicare

for beneficiaries in the cohort of interest.

*Data structure should be one row per denosumab claim.

*Part B claims should be identified as those associated with line item HCPCS code = J0897, and

*Part D claims should be identified using Part D SEvent file claims with National Drug Code = 55513-0710-01 or 55513-0710-21

**NB: ENSURE UPDATED HCPCS AND NDC CODES ARE EXPLORED FOR YOUR DATASET AND STUDY PERIOD.

**NB: SEE CMS/RESDAC DOCUMENTATION FOR MOST RECENT PART D EVENT PART B CLAIMS AND LINE ITEM FILE DETAILS.

*This dataset must contain the following variables:

a. ID = unique beneficiary identifier

b. index_date = date of first denosumab dispensing

c. death_date = date of death for the beneficiary

c. NH_date = first date of long-term nursing home

care receipt for the beneficiary (if

restricting to community-dwelling

beneficiaries)

d. claim_date = date of claim (both Part B & Part D claims)

e. service_count = service count variable from Part B line

items (Part B claims only)

************************************************************************/

***********************************************;

*STEP 1: DELETE INELIGIBLE CLAIMS

a. Exclude Part B claims where service_count=120

b. Exclude all claims after death

c. Exclude all claims after NH entry

(if focusing on community dwelling beneficiaries);

***********************************************;

**proc** **sort** data=DMAB_CLAIMS;

by id claim_date;

**run**;

**data** DMAB_CLAIMS_MASTER;

set DMAB_CLAIMS;

if service_count=**120** then delete;

if claim_date > death_date then delete;

if claim_date > NH_date then delete;

**run**;

/*Note: Code assumes beneficiaries with NH_date > death_date are excluded*/

***********************************************;

* STEP 2: MODIFY DATA STRUCTURE

a. Use proc expand to transpose long dataset

to wide dataset - mapping out claims

in 3 claim increments

b. Calculate time between claims

in order to apply "121 day" rules

c. Create time variable to denote claim number

***********************************************;

**proc** **sort**

data=DMAB_CLAIMS_MASTER

out= DMAB_CLAIMS_DEDUP

nodupkey;

by id claim_date;

**run**;

**proc** **expand** data=DMAB_CLAIMS_DEDUP(keep= ID claim_date)

out=DMAB_CLAIMS_EXPAND;

convert claim_date = claim_date2 / transformout = (lead **2**) method=none;

convert claim_date = claim_date1 / transformout = (lead **1**) method=none;

convert claim_date = claim_date0 / transformout = (lag **1**) method=none;

by ID;

**run**;

**data** DMAB_CLAIMS_EXPAND_A;

set DMAB_CLAIMS_EXPAND;

by ID;

time = time+**1**;

interval0 = claim_date - claim_date0; /*Prior interval*/

interval1 = claim_date1 - claim_date; /*Current interval*/

interval2 = claim_date2 - claim_date1; /*Next interval*/

**run**;

***********************************************;

* STEP 3: FLAG INDIVIDUALS TO EXCLUDE

a. Flag people with 3 consecutive claims

< 121 days apart

b. Exclude due to off label malignancy

dosing or potential claims errors

***********************************************;

**data** DMAB_CLAIMS_EXPAND_B;

set DMAB_CLAIMS_EXPAND_A;

fl_lt121 = **0**;

if interval0 < **121** and

interval1 < **121** and

interval2 < **121** and

interval1 ne **.**

then fl_lt121 = **1**;

**run**;

/*Retain only claims for those without fl_lt121*/

**proc** **sql**;

create table DMAB_CLAIMS_FORCLEANING

as select *

from DMAB_CLAIMS_EXPAND_B

where ID not in

(select ID

from DMAB_CLAIMS_EXPAND_B

where fl_lt121 = **1**);

**quit**;

***********************************************;

* STEP 4: IDENTIFY INDEX DATE

a. Define index date (first denosumab claim)

and apply cleaning rules for index date

b. Remove old index claims if needed to be

cleaned

***********************************************;

/*Defining index date*/

**data** DMAB_CLAIMS_FORCLEANING_A;

set DMAB_CLAIMS_FORCLEANING;

by ID;

if first.ID then do;

index_date=**.**;

end;

/*Cleaning up the index date and keeping those with only one claim*/

if first.ID and (interval1 >= **121** or interval1 =**.**)

then index_date=claim_date;

else if first.ID and **.**< interval1 < **121** and interval2 =**.**

then do;

index_exclude=**1**;

index_date=claim_date1;

end;

else if first.ID and **.**< interval1 < **121** and interval2 >= **121**

then do;

index_exclude=**2**;

index_date=claim_date1;

end;

else if first.ID and **.**< interval1 < **121** and **.**< interval2 < **121**

and **.**< interval1+interval2 < **121**

then do;

bene_flag1=ID

time_flag1=time;

index_exclude=**3**;

index_date=claim_date2;

end;

else if first.ID and **.**< interval1 < **121** and **.**< interval2 < **121**

and **.**< interval1+interval2 >= **121**

then do;

bene_flag2=ID;

time_flag2=time+**1**;

index_date=claim_date;

end;

if ID=bene_flag1 and time=time_flag1+**1** then index_exclude=**3**;

if ID=bene_flag2 and time=time_flag2 then index_exclude=**4**;

format index_date date9.;

**run**;

/*Check for people who had 3 claims < 121 days apart*/

**proc** **sort**

data=DMAB_CLAIMS_FORCLEANING_A(where= (fl_index_cleaning ne **.**))

nodupkey

out=DMAB_CLAIMS_FORCLEANING_B(keep = ID fl_index_cleaning);

by ID;

**run**;

***********************************************;

* STEP 5: CLEAN INDEX CLAIM (I.E., FIRST DMAB CLAIM)

a. Flag patients who need index claims fixed

b. Exclude claims that occurred prior to new index date

For the example beneficiary below, claim #2 will become the

index claim and claim #1 will be deleted

DMAB claim #1: 13-AUG-2019

DMAB claim #2: 23-AUG-2019 (+ 10 days after claim #1)

DMAB claim #3: 31-MAR-2020 (+221 days after claim #2)

***********************************************;

**proc** **sql**;

create table DMAB_CLAIMS_FORCLEANING_C as

select *,

max(index_exclude) >= **1** as i_index_exclude,

count(*) as total_original

from DMAB_CLAIMS_FORCLEANING_A

/*Note: use '_A', not '_B' dataset*/

group by ID

order by ID, claim_date;

**quit**;

/*REMOVE DMAB CLAIMS THAT OCCURRED BEFORE THE "push forward" DATE*/

**data** DMAB_CLAIMS_FORCLEANING_D;

set DMAB_CLAIMS_FORCLEANING_C;

if index_exclude in (**1**,**2**,**3**,**4**) then delete;

**run**;

/*NOTE: WE ARE NOT DELETING BENEFICIARIES ALL-TOGETHER;

WE JUST DELETE THE "OLD" INDEX DMAB CLAIMS*/

***********************************************;

* STEP 6: RESTRUCTURE DATASET

a. Lead and lag claim dates after cleaning index claim

b. Create new time variable to denote updated claim number

c. Flag intervals of claims 121 days apart again

***********************************************;

/*Update data structure again after cleaning index claim*/

**proc** **expand**

data=DMAB_CLAIMS_FORCLEANING_D

(keep= ID time claim_date index_date

i_index_exclude total_original)

out=DMAB_CLAIMS_FORCLEANING_E ;

convert claim_date = claim_date2 / transformout = (lead **2**) method=none;

convert claim_date = claim_date1 / transformout = (lead **1**) method=none;

convert claim_date = claim_date0 / transformout = (lag **1**) method=none;

by ID;

**run**;

/*Identify intervals of 121 days again*/

**data** DMAB_CLAIMS_FORCLEANING_F;

set DMAB_CLAIMS_FORCLEANING_E;

by ID;

time = time+**1**;

interval0 = claim_date - claim_date0; /*Prior interval*/

interval1 = claim_date1 - claim_date; /*Current interval*/

interval2 = claim_date2 - claim_date1; /*Next interval*/

if interval0 >= **121** and **.**< interval1 < **121** and interval2 >= **121**

then claim_exclude=**1**;

else if interval0 >= **121** and **.**< interval1 < **121** and

**.**< interval2 < **121** and **.**< interval1+interval2 < **121**

then claim_exclude=**2**;

else if **.**<interval0 < **121** and **.**< interval1 < **121**

then claim_exclude=**3**;

else if interval0 >= **121** and **.**< interval1 < **121** and interval2 =**.**

then claim_exclude=**4**;

if (claim_exclude=**3** and interval2=**.**) or (claim_exclude=**4**)

then claim_end_exclude=**1**;

if claim_end_exclude ne **1** and claim_exclude in (**1**,**2**,**3**)

then claim_notend_exclude=**1**;

**run**;

***********************************************;

* STEP 7: CLEAN LAST DMAB CLAIM

a. Flag patients who need last DMAB claims fixed

b. Exclude relevant claims

***********************************************;

/*Flagging patients who need end claims cleaned*/

**proc** **sql**;

create table DMAB_CLAIMS_FORCLEANING_G as

select *,

max(claim_end_exclude) as i_end_exclude,

max(claim_notend_exclude) as i_notend_exclude

from DMAB_CLAIMS_FORCLEANING_F

group by ID

order by ID, claim_date;

**quit**;

/*Exclude claims*/

**data** DMAB_CLAIMS_FORCLEANING_H

(rename= (claim_date=new_claim_date

index_date=new_index_date));

set DMAB_CLAIMS_FORCLEANING_G;

if claim_exclude in (**1**,**2**,**3**,**4**) then delete;

if i_end_exclude=**.** then i_end_exclude=**0**;

keep ID claim_date index_date i_index_exclude i_end_exclude;

**run**;

**proc** **sort** data=DMAB_CLAIMS_FORCLEANING_H;

by ID new_claim_date;

**run**;

***********************************************;

* STEP 8: RESTRUCTURE DATASET

a. Re-calculate dispensing number and intervals

b. Impose 1 year follow-up criteria

***********************************************;

**proc** **expand**

data=DMAB_CLAIMS_FORCLEANING_H

out=DMAB_CLAIMS_FORCLEANING_I;

convert new_claim_date = claim_date1 / transformout = (lead **1**) method=none;

by ID;

**run**;

**data** DMAB_CLAIMS_CLEANED;

set DMAB_CLAIMS_FORCLEANING_I;

by ID;

dispen=time+**1**;

new_days = claim_date1 - new_claim_date;

drop time claim_date1;

**run**;

/*END OF PROGRAM*/
